# Granulations

**Published:** 1886-04-20

**Authors:** 


**Granulations.**—Germicide: For granulations, swellings, buboes, erysipelas the following is of great utility: Iodide of ammonia, 2 drachms ; iodide of cadimum, bromide of cadimum, of each half a drachm ; ozone ointment, from a half to one ounce. Mix. Apply three times a day with a camel's hair brush.

